# Clinical characteristics and molecular epidemiology of hepatitis E in Shenzhen, China: a shift toward foodborne transmission of hepatitis E virus infection

**DOI:** 10.1038/emi.2017.107

**Published:** 2017-12-20

**Authors:** Siddharth Sridhar, Simon KF Lo, Fanfan Xing, Jin Yang, Haiyan Ye, Jasper FW Chan, Jade LL Teng, Chuan Huang, Cyril CY Yip, Susanna KP Lau, Patrick CY Woo

**Affiliations:** 1 State Key Laboratory of Emerging Infectious Diseases, Hong Kong, China; 2 Research Centre of Infection and Immunology, The University of Hong Kong, Hong Kong, China; 3 Carol Yu Centre for Infection, The University of Hong Kong, Hong Kong, China; 4 Department of Microbiology, Li Ka Shing Faculty of Medicine, The University of Hong Kong, Hong Kong, China; 5 Department of Clinical Microbiology and Infection Control, The University of Hong Kong – Shenzhen Hospital, Shenzhen 518053, China; 6 Collaborative Innovation Center for Diagnosis and Treatment of Infectious Diseases, The University of Hong Kong, Hong Kong, China

**Keywords:** China, hepatitis E, molecular epidemiology, non-alcoholic fatty liver disease

## Abstract

Hepatitis E virus (HEV) is the most common cause of acute viral hepatitis in China. Recently, a shift in molecular epidemiology from hepatitis E genotype 1 (HEV-1) to hepatitis E genotype 4 (HEV-4) has been observed in Northern China, marking a switch from human-to-human transmission to zoonosis. However, similar data from cities in Southern China are lacking. This observational study of human hepatitis E cases in Shenzhen, a metropolitan city in the Pearl River Delta region, aimed to describe the clinical features and molecular epidemiology of hepatitis E in Southern China. Over a 55-month period, we identified 20 patients with acute hepatitis E. Most were middle-aged men, and 50% of patients had concomitant liver disease, of whom 70% were identified to have non-alcoholic fatty liver disease; such patients had a trend toward higher liver enzymes. Quantitative real-time RT-PCR using archived serum samples showed that 12 patients had hepatitis E viremia at presentation. Sequencing of the RNA-dependent RNA polymerase gene was performed for five of these patients, and phylogenetic analysis revealed that these five HEV isolates belonged to subgenotype 4b and were clustered with swine HEV isolates from Southern China. Combined with other studies showing similar findings, this suggests that the molecular epidemiology of hepatitis E in China is evolving toward low-level endemicity driven by foodborne transmission from seafood or pork products. The importance of concomitant liver disease, in particular non-alcoholic fatty liver disease, as a risk factor for severe hepatitis E requires further study.

## INTRODUCTION

Hepatitis E virus (HEV) is estimated to cause 20 million infections worldwide annually.^1^ Hepatitis E can cause fulminant hepatic failure and chronic infection in immunocompromised patients.^2^ In China, hepatitis E has overtaken hepatitis A in terms of both incidence and mortality rates over the past decade.^3^ This trend is driven by a combination of factors: increasing vaccination coverage against hepatitis A, improving sanitation, and an epidemiological shift in the transmission patterns of hepatitis E.

Four genotypes of hepatitis E have been commonly associated with human infection. Genotypes 1 and 2 are confined to human populations, whereas genotypes 3 and 4 circulate between swine and human populations.^4^ Historically, most cases of hepatitis E in China in the twentieth century have been caused by genotype 1 HEV (HEV-1), which causes large water-borne outbreaks driven by person-to-person transmission.^5, 6^ However, over the past two decades, HEV-1 appears to have been replaced by genotype 4 HEV (HEV-4) as the most common genotype causing human infection (>80% of cases) in Northern China.^6^ HEV-4 is endemic in swine populations across China and is transmitted to humans who consume undercooked pork. This shift in molecular epidemiology has been well studied in Northern and North-eastern China.^7, 8^

Although studies have documented the presence of HEV-4 in pigs in Southern China,^9, 10^ there is a paucity of data on the epidemiology and clinical characteristics of human hepatitis E in this region. This is a significant shortcoming given the high population density of the heavily industrialized Pearl River Delta region of Southern China. The objective of this study was to profile the molecular epidemiology and clinical characteristics of hepatitis E in Shenzhen, a Southern Chinese metropolitan city with a large immigrant population from other parts of China. Using a phylogenetic analysis, we aimed to test the hypothesis that zoonotic transmission is a common route of acquisition of hepatitis E in Southern China.

## MATERIALS AND METHODS

### Patients

This was a retrospective study conducted between 1 July 2012 and 31 January 2017 at The University of Hong Kong – Shenzhen hospital. This 1400-bed multi-specialty hospital was established in 2012 and provides primary to tertiary medical services to the residents of Shenzhen city in both inpatient and outpatient settings. Shenzhen is a special economic zone with an estimated population of nearly 11 million people, including a large migrant population from other regions in China. Patients admitted as inpatients with acute hepatitis syndrome to The University of Hong Kong – Shenzhen hospital were evaluated for hepatitis E infection. Patients were defined to have hepatitis E if they presented with a clinical syndrome of acute hepatitis and tested positive for hepatitis E immunoglobulin M (IgM) antibodies in serum (using the kit cutoff criteria). Clinical details, travel history, epidemiological risk factors for hepatitis E and liver function test results for these patients were retrieved. Ethics approval for this study was provided by the Institutional Review Board of The University of Hong Kong – Shenzhen hospital.

### Hepatitis E serology

Enzyme-linked immunosorbent assay (ELISA) testing for anti-HEV IgM was performed using the Wantai HEV-IgM ELISA (Wantai, Beijing Wantai Biological Pharmacy enterprise Co., Ltd, Beijing, China). This kit has been reported to have a diagnostic specificity for acute hepatitis E of >99%.^11^ The microtiter plates used for ELISA testing were processed in a Uranus AE280 automated analyzer (Aikang Medtech Co. Ltd, Shenzhen, China). All serum samples were stored in an ultra-low freezer after serological testing.

### HEV viral load testing

If the specimen quantity was sufficient, serum samples testing positive for anti-HEV IgM antibodies were subjected to HEV real-time reverse transcriptase PCR (RT-PCR) using primers and conditions targeting the HEV ORF3 gene.^12^ Viral RNA was extracted from the serum samples using the EZ1 Virus Mini Kit v2.0 (Qiagen, Hilden, Germany). Real-time one-step RT-PCR assays were also performed using the QuantiNova Probe RT-PCR Kit (Qiagen) in a LightCycler 96 Real-Time PCR System (Roche Diagnostics, Rotkreuz ZG, Switzerland). Each 20 μL-reaction mix contained 1 × QuantiNova Probe RT-PCR Master Mix, 1 × QN Probe RT-Mix, 0.8 μM forward and reverse primers, 0.2 μM probe and 5 μL of template RNA. The reactions were incubated at 45 °C for 10 min and 95 °C for 5 min, followed by 50 cycles at 95 °C for 5 s and 55 °C for 30 s.

### HEV sequencing

Extracted RNA was eluted in 60 μL of AVE buffer (Qiagen). RT for the sequencing reaction was performed using the SuperScript III Kit (Invitrogen, USA); the reaction mixture (10 μL) contained RNA, first-strand buffer (50 mM Tris-HCl pH 8.3, 75 mM KCl, 3 mM MgCl_2_), 5 mM DTT, 50 ng of random hexamers, 500 μM of each dNTPs and 100 U Superscript III reverse transcriptase. The mixtures were incubated at 25 °C for 5 min, followed by 50 °C for 60 min and 70 °C for 15 min.

PCR and sequencing of the HEV RNA-dependent RNA polymerase (RdRp) gene were performed using the MJ-C primers and conditions as previously described.^13^ These primers were designed from the multiple alignment of 37 HEV genome sequences, covering the four genotypes HEV-1–HEV-4. All PCR products were gel-purified using the QIAquick Gel Extraction Kit (Qiagen). Both strands of the PCR products were sequenced twice with an ABI Prism 3730xl DNA Analyzer (Applied Biosystems, Foster City, CA, USA) using the two PCR primers.

Using the sequences obtained from the method above, two new nested primer pairs were designed to detect HEV sequences in serum specimens testing negative using the MJ-C primers. The new outer primers were LPW36103 (5′- CTTGGTTCCGCGCTATTGA-3′) and LPW36106 (5′- GACCCAAGCAGAACGAACA-3′), whereas the inner primers were LPW36104 (5′- CAAGCAGAACGAACAAGATGG-3′) and LPW36105 (5′- TTGGTTCCGCGCTATTGAA-3′). Two rounds of PCR amplification were performed in 50 μL of PCR mixture containing PCR buffer (10 mM Tris-HCl pH 8.3, 50 mM KCl, 2 mM MgCl_2_), 200 μM of each dNTP, 10 μM external or internal sense and anti-sense primers, 1.0 U Taq polymerase (Applied Biosystems), water and the template. The PCR cycling conditions for each round were as follows: hot start at 95 °C for 10 min, followed by 30 cycles of 94 °C for 1 min, 48 °C for 1 min and 72 °C for 1 min with a final extension at 72 °C for 10 min in an automated thermal cycler (Applied Biosystems). Standard precautions were taken to avoid PCR contamination, and no false-positive signal was observed in the negative controls.

### Phylogenetic analysis

A phylogenetic tree was constructed using the maximum-likelihood method in MEGA 7.0. A Kimura two-parameter model with gamma distribution and invariant sites (G+I) was used for phylogenetic inference.

### Statistical analysis

The statistical analyses included Student’s *t*-test for two samples, Wilcoxon signed-rank test and linear regression modeling with the calculation of *r*-squared as appropriate.

## RESULTS

### Hepatitis E cases

**Figure 1:**
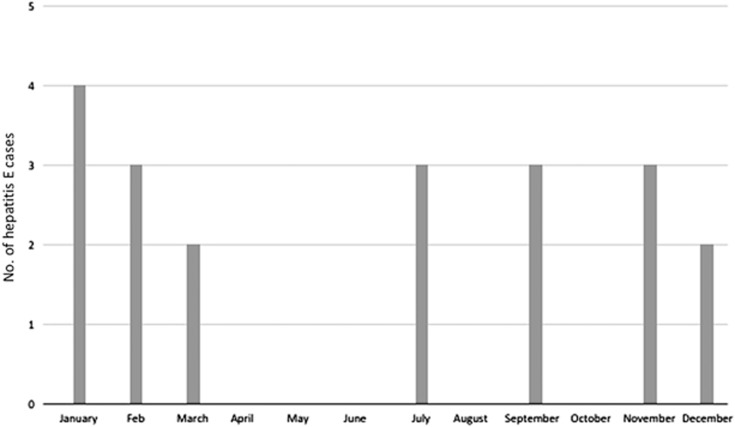
Monthly variation in the incidence of acute hepatitis E cases.

Over the 55-month study period, which was conducted since the introduction of inpatient services at The University of Hong Kong – Shenzhen hospital, 20 patients were diagnosed with acute hepatitis E. Their clinical and demographic features are summarized in Table 1. The majority of patients were middle aged, with 80% of patients aged >35 years. Men were affected disproportionately in a 3:1 ratio. Three of the patients recalled consuming undercooked seafood approximately 1 month before hepatitis onset. A definite history of consuming undercooked pork products could not be elicited from any of the patients. Patients presented within 2 days to 4 weeks of symptom onset. None of the five female patients were pregnant. Seventeen cases (85%) occurred between September and March (Figure 1).

All patients had elevated alanine aminotransferase (ALT) with a range between 129 and 6650 U/L (median: 1072 U/L). Serum alkaline phosphatase was measured in 13 patients and was elevated >110 U/L in 11 patients (85% median: 193 U/L). The median bilirubin was 34 μmol/L; 65% of patients had an elevated bilirubin (> 23 μmol/L) at the time of presentation to our center. Elevation in prothrombin time was observed in five patients. None of the patients had renal impairment or extrahepatic manifestations. All patients had self-limited hepatitis without decompensation and had improvement in liver function tests with supportive management. None of the cases required liver transplantation.

A diagnosis of concomitant non-alcoholic fatty liver disease (NAFLD) was made in 7 patients (35%) and represented the most common underlying liver disease in this patient cohort. In addition, two patients were chronic hepatitis B carriers, and one had alcoholic fatty liver disease (AFLD). In total, 13/20 patients had sufficient serum available for testing anti-HAV IgM and anti-HCV antibodies, and all 13 were negative for these two serological tests. Overall, patients with underlying liver disease (NAFLD, HBV carriage or AFLD) had higher mean total bilirubin and ALT levels compared with those with no concomitant chronic liver condition (71 vs. 42 μmol/L and 2290 vs. 1232 U/L, respectively) although this finding did not reach statistical significance (*P*-values by Student’s *t*-test: 0.229; 0.132, respectively). Similarly, a trend for higher mean ALT was observed among the NAFLD patients compared with patients with no underlying liver disease (1814 vs. 1232 U/L; *P-*value by Student’s *t*-test: 0.292). Two patients had underlying immunosuppression. One patient was immunosuppressed owing to systemic lupus erythematosus requiring hydroxychloroquine, prednisolone and mycophenolate mofetil, whereas the other had neutropenia. Both patients had normal bilirubin but had elevated ALT to 707 and 2771 U/L.

### HEV RT-PCR and viral load

Of the 20 patients, 12 had residual sera available for HEV RT-PCR and viral load measurement. All 12 patients had diagnostic testing performed within the first 2 weeks of clinical disease. Of these 12 patients, 8 patients had detectable hepatitis E RNA in one or more serum specimens. Their clinical characteristics and viral loads are summarized in Table 2. Viral loads in the positive sera ranged from 1.31 × 10^4^ to 8.29 × 10^6^ genome copies/mL of serum. Patients with HEV viremia had a trend toward higher liver function tests compared with hepatitis E patients without viremia; the median ALT was 1928 U/L in the former group and 600 U/L in the latter group, but this finding did not attain statistical significance (*P-*value>0.1 by the Wilcoxon signed-rank test). There was no correlation between viral loads and alanine aminotransferase at admission upon linear regression analysis (*P-*value=0.13; *r*-squared=0.339).

### Genotyping and phylogenetic analysis

**Figure 2:**
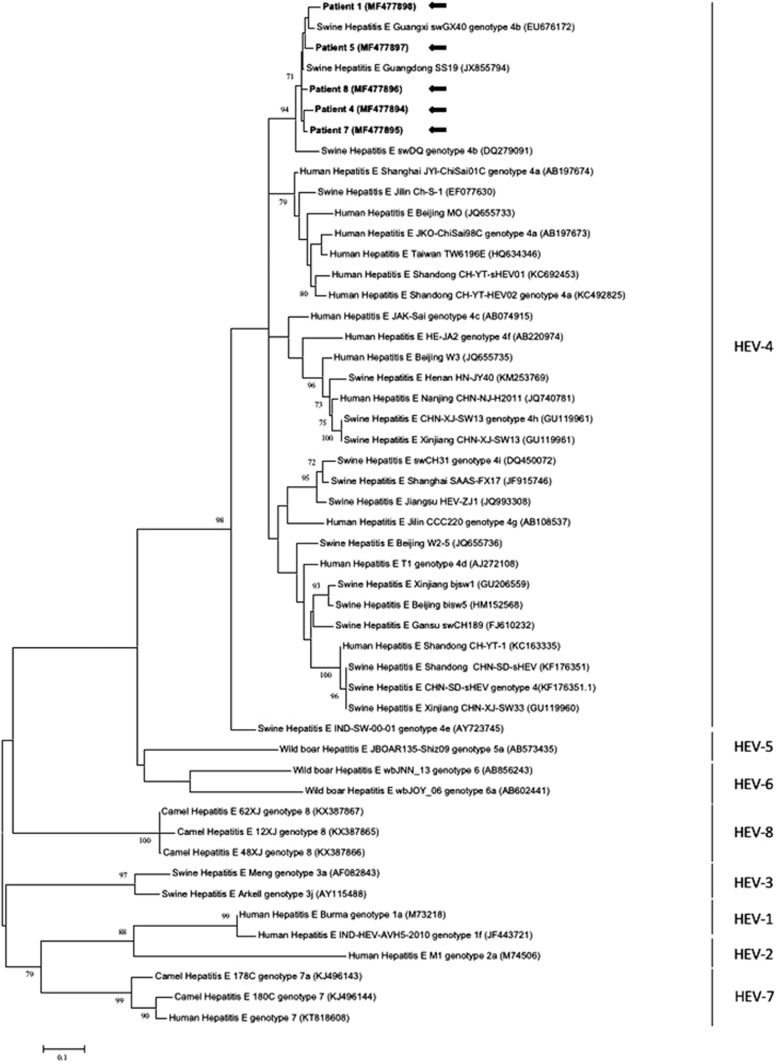
Phylogenetic analyses of the partial RdRp region of the five human HEV isolates sequenced in this study (patients 1, 4, 5, 7 and 8 as per Table 2) and other HEV genotypes. The tree was constructed based on the maximum-likelihood method using the Kimura two-parameter model with invariant sites and gamma distributed with invariant sites (G+I). The bootstrap analysis was performed with 1000 replicates. Bootstrap values <70% are not shown. The analysis included 257 nucleotide positions (nucleotide position 4274–4530 numbered with reference to GenBank sequence M73218). GenBank accession numbers are shown in parentheses. The scale bar indicates the estimated number of substitutions per site. Hepatitis E virus (HEV).

Serum specimens testing positive for HEV RNA were subjected to sequencing of a segment of the HEV RdRp gene, followed by phylogenetic comparison with other human and swine HEV isolates obtained from China (Figure 2). Five out of the 12 patients had sufficient sequencing data available for accurate genotype designation. All five HEV isolates belonged to subgenotype 4b and were clustered closely with swine HEV isolates from Guangzhou (SS19) and Guangxi provinces (swGX40) of Southern China. The percentage of nucleotide identities between the individual isolates and the closest complete HEV-4b genome is depicted in Table 3, which shows that the isolate from patient 1 was the most distant, sharing 93–94% nucleotide identity with the other isolates. One of the patients had a definite history of consumption of raw oysters approximately 1 month before symptom onset (patient 8 in Table 2). Of the five patients confirmed to have HEV-4b infection, three were migrants from Northern China (two from Beijing and one from Anhui province), but they had not returned to their home provinces within 3 months of symptom onset.

## DISCUSSION

Owing to rapid industrialization and socioeconomic development, incidence and mortality of hepatitis A in China have undergone sharp declines, leaving hepatitis E as the most common cause of acute viral hepatitis.^3^ Such an epidemiological shift is likely driven by ongoing zoonotic foodborne transmission of HEV, which has been demonstrated in swine populations across the country. In this systematic investigation of human hepatitis E infections in Shenzhen, we noted several novel features of hepatitis E in a key population center of the highly industrialized Pearl River Delta region of southern China with a large migrant population. Most patients with acute hepatitis E in this study were middle-aged men, an epidemiological feature commonly associated with autochthonous hepatitis E in developed regions, in contrast to hepatitis E hyperendemic regions, where younger individuals are typically affected. We did not note any pregnant women presenting with fulminant hepatitis E during the study period; this entity is typically rare in developed regions. Both these features suggest that urban centers in Southern China have shifted from hepatitis E hyperendemicity to a low-level autochthonous form of hepatitis E.

An analysis of the monthly incidence of cases showed that 85% of cases occurred during the cooler months between September and March. This winter peak has also been reported in another recent Chinese study.^14^ The HEV prevalence in swine samples was observed to peak during September–October in Southwestern China,^15^ which may account for a spike in human cases during the ensuing winter months, although the exact cause of this seasonal variation remains elusive.

Concomitant liver disease was noted in half the patients presenting with acute hepatitis E. This study found a trend toward higher ALT and bilirubin at presentation in such patients. Strikingly, radiological evidence of NAFLD was noted in 35% of all patients with hepatitis E. Certain forms of chronic liver disease such as hepatitis B-related cirrhosis are known to be associated with severe hepatitis E. The effects of underlying NAFLD and non-alcoholic steatohepatitis on hepatitis E clinical severity require urgent further investigation in view of the increasing rates of NAFLD in China.^16^

Three patients in this cohort had consumed undercooked seafood approximately 4 weeks prior to presentation (that is, within the incubation period of hepatitis E), and one was confirmed to be infected with HEV-4b. Although foodborne hepatitis E is most often associated with the consumption of undercooked pork, the prevalence of HEV in pork samples from local markets in Shenzhen is not known. Another potential zoonotic source of hepatitis E in China is rabbits; both laboratory and farmed rabbits have been described to carry HEV variants in China,^17, 18^ and transmission to humans has been described.^19^ HEV (subgenotypes 4b and 4d) has been detected in bivalve shellfish from the Bohai Gulf of China,^20^ likely concentrated in filter feeders from contaminated pig farm effluent. Sewage from livestock farms in Shenzhen has been documented to contain HEV-4 RNA.^21^ It is likely that most patients in this study acquired the infection via the consumption of pork products; however, based on the epidemiological history, transmission from other zoonotic sources remains possible. Other modes of transmission, such as household transmission and blood-borne transmission, cannot be ruled out completely.

All five HEV isolates obtained in this study belonged to subgenotype 4b, which has previously been shown to be the most common HEV subgenotype infecting humans in Eastern China,^22^ but it appears to be an uncommon cause of human hepatitis in Northern China where HEV-4a and HEV-4d predominate.^23, 24^ There are no known differences in pathogenicity or source of infection between different HEV subgenotypes. As HEV-4 predominantly circulates in swine (and may contaminate shellfish farms via effluent from pig farms), the fact that all the isolates in this study are HEV-4 indicates that hepatitis E in Southern China has evolved to a foodborne disease typical of an area of low endemicity. Given that a previous analysis of human hepatitis E cases in Southern China between 1994 and 1998 indicated that all human HEV isolates were HEV-1,^25^ this study provides confirmation that the Chinese epidemiological shift from HEV-1 to HEV-4 over the past two decades has affected Southern China as well.

Because this study was retrospective, not all patients had enough sera available for a detailed exclusion of other causes of viral hepatitis such as hepatitis A or hepatitis C. Furthermore, convalescent sera were not available for detecting HEV IgG seroconversion as a confirmation of diagnosis. However, we used the Wantai HEV IgM assay in this study, which has a reported diagnostic specificity of >99% for hepatitis E. The positive predictive value of this assay in an endemic area would be expected to be quite high.

Another limitation of this study was the small sample size; furthermore, not all patient sera were available for detailed virological analysis by RT-PCR and sequencing. Furthermore, HEV RNA can degrade in stored archived sera over time even when stored in ultralow freezers; therefore, retrospective RT-PCR may not accurately measure the actual viral load in the sample at the time of collection. This might affect the statistical analysis of the correlation between viral load and ALT at presentation. Another limitation of this study was the lack of long-term follow-up data for some of the patients. This is particularly important for the two immunocompromised patients in this cohort because hepatitis E infections can become chronic in immunocompromised patients.^26^

In total, 50% of the patients included in this study were originally from provinces in Northern China; therefore, the epidemiological trends observed in this study may not be unique to Southern China. However, for the five patients with genotyping results, although three were originally from Beijing and Anhui province, they had all been infected with HEV subtype 4b rather than subtypes 4a or 4d, which predominated in Northern China.^15, 27^ This indicates that the genotyping results of this study are suggestive of current trends in Southern China.

In summary, this study provides evidence of a major epidemiological shift in hepatitis E in Southern China driven by genotype switch, which has an observable effect on the clinical characteristics of the disease in the region. The role of underlying liver disease, particularly NAFLD, in exacerbating hepatitis E must be elucidated further.

## Figures and Tables

**Table 1:** Clinical features of hepatitis E patients identified in this study

*Gender*	
Male (*N*, %)	15; 75
Female (*N*, %)	5; 25
	
Age (median; range in years)	42.5; 28–66
	
*Underlying liver disease (*N, *%)*	
Non-alcoholic fatty liver disease	7; 35
Alcoholic fatty liver disease	1; 5
Hepatitis B carriage	2; 10
	
Underlying immunosuppression (*N*, %)	2; 10
*Travel outside China 2–6 weeks preceding symptom onset* (N, *%*)	3; 15
Thailand (*N*)	1
Vietnam (*N*)	1
Japan (*N*)	1
	
*Presenting symptom* (N, *%)*	
Fever	6; 30
Abdominal pain	6; 30
Diarrhea	1; 5
Anorexia	15; 75
Nausea	8; 40
Tea-colored urine	11; 55

**Table 2:** Clinical and virological features of patients with detectable virus in the bloodstream

**Patient**	**Age/sex**	**History of consumption of shellfish**	**Underlying liver disease**	**Bilirubin (μmol/L) at admission**	**ALT (U/L) at admission**	**Viral load (copies/mL of serum); time since symptom onset**	**Genotype**
1	66/M	No	NAFLD	59.3	3645	8.29 × 10^6^; 3 days	4b
2	50/M	No	NAFLD	115.4	2991	1.31 × 10^4^; ~2 weeks	NA
3	55/M	Yes	AFLD	68.6	785	1.42 × 10^4^; 15 days	NA
4	40/F	No	None	11.7	707	1.79 × 10^4^; 12 days	4b
5	43/F	No	Hepatitis B carrier	16.5	2771	1.37 × 10^5^; 11 days	4b
6	36/M	No	NAFLD	8.5	616	4.23 × 10^4^; NA	NA
7	44/M	No	None	30.5	2189	4.99 × 10^6^; 10 days	4b
8	39/M	Yes	None	35.9	1668	1.43 × 10^4^; 7 days	4b

Abbreviations: alcoholic fatty liver disease (AFLD); alanine aminotransferase (ALT); female (F); male (M); not available (NA); NAFLD, non-alcoholic fatty liver disease (NAFLD).

**Table 3:** Percentage of nucleotide identities between HEV-4b isolates from individual patients and closest complete HEV-4b genomes

	**Patient 1**	**Patient 4**	**Patient 5**	**Patient 7**	**Patient 8**	**SS19**	**swGX40**
Patient 1		93	94	93	94		96
Patient 4	93		95	97	96	97	
Patient 5	94	95		95	96	97	
Patient 7	93	97	95		96	97	
Patient 8	94	96	96	96		98	

Abbreviation: hepatitis E virus (HEV). The GenBank accession number of SS19 is JX855794.1 and that of swGX40 is EU676172.2.
